# Laxative Use in the Community: A Literature Review

**DOI:** 10.3390/jcm10010143

**Published:** 2021-01-04

**Authors:** Barry L. Werth, Sybèle-Anne Christopher

**Affiliations:** Susan Wakil School of Nursing and Midwifery, Faculty of Medicine and Health, The University of Sydney, Camperdown, NSW 2006, Australia; sybele.christopher@sydney.edu.au

**Keywords:** laxatives, constipation, adults, prevalence, utilisation

## Abstract

Laxatives are widely available without prescription and, as a consequence, they are commonly used for self-management of constipation by community-dwelling adults. However, it is not clear to what extent laxatives are used. Nor is it clear how laxatives are chosen, how they are used and whether consumers are satisfied with their performance. This review of published literature in the last 30 years shows the prevalence of laxative use in community-dwelling adults varied widely from 1% to 18%. The prevalence of laxative use in adults with any constipation (including both chronic and sporadic constipation) also varied widely from 3% to 59%. Apart from any geographical differences and differences in research methodologies, this wide range of estimated prevalence may be largely attributed to different definitions used for laxatives. This review also shows that laxative choice varies, and healthcare professionals are infrequently involved in selection. Consequently, satisfaction levels with laxatives are reported to be low and this may be because the laxatives chosen may not always be appropriate for the intended use. To improve constipation management in community and primary healthcare settings, further research is required to determine the true prevalence of laxative use and to fully understand laxative utilisation.

## 1. Introduction

Laxatives accelerate or induce defecation [1] and are often used in the management of constipation in the community [2]. Constipation, a common community problem globally [3,4], is frequently self-diagnosed and self-managed by community-dwelling adults [5]. Constipation represents a substantial cost in the community [6], particularly chronic constipation which is usually defined by a set of clinical symptoms known as the Rome criteria [7]; these criteria have been revised several times since their introduction in 1994 as Rome I criteria. Constipation also includes both chronic and sporadic constipation [8]. Most adults attempt to self-manage their constipation before consulting a healthcare professional [9]. Self-management often includes the use of laxative products, most of which may be purchased in pharmacies and elsewhere without prescription. However, failures in the self-management of constipation are frequent and lead to additional costs which add considerably to the financial burden of constipation in the community [10].

Laxative pharmaceutical products available without prescription are generally referred to as over-the-counter (OTC) laxatives. Classification of laxatives is based on the mode of action and the four main classes of OTC laxatives are bulk-forming laxatives, softeners/lubricants, contact/stimulant laxatives and osmotic laxatives [1]. This classification is commonly used worldwide and is incorporated in the World Health Organization’s list of drugs for constipation as defined by the Anatomical Therapeutic Classification (ATC) [11]. For optimal management of constipation, healthcare professionals working in primary healthcare settings need to understand the extent of OTC laxative use in the community and how laxatives are used by community-dwelling adults. Because OTC laxatives are widely available without prescription, it is not clear which laxative agents are being used, why and how they are selected, and for what purpose they are used. OTC laxatives are intended for use in the management of constipation although they are sometimes used by consumers for other purposes such as weight loss [12]. In managing constipation, OTC laxatives may be used in two ways—either for treatment or for prevention of constipation [13]. Treatment of constipation refers to the use of a laxative to relieve constipation symptoms. Prevention of constipation refers to use of a laxative to prevent the symptoms of constipation from occurring. In the context of constipation management, it is also important to understand consumer satisfaction regarding OTC laxative effectiveness [14]. Although laxatives feature prominently in constipation management, rigorous scientific evidence for their efficacy is scarce because most OTC laxatives have been in use for several decades [15]. Nevertheless, therapeutic outcomes of OTC laxative usage in the community are not necessarily reflected in clinical trials [16].

The aims of this review are to report the prevalence of laxative use, and to report on the choice and utilisation of laxatives as well as satisfaction with laxatives, in community-dwelling adult populations.

## 2. Methods

Relevant literature published in the period 1989 to 2019 was located using Medline and Embase databases. Search terms in various combinations using the AND Boolean operator were applied using keywords and combined terms to refine the search process: “constipation”, “laxative”, “prevalence”, “survey”, “adults”, “population”. In addition to electronic database searches, the search strategy employed for this review also included the “ancestry approach” [17] where the references of yielded articles were examined for relevant studies reporting laxative use in the community.

The search was limited to population-based studies which reported or described the prevalence of laxative use or laxative utilisation in community-dwelling adults. The search was also limited to English language articles. Studies were excluded if the sample size was fewer than 100 participants or if subpopulations such as older adults or only one gender were used.

Articles were identified and screened for their eligibility according to the inclusion and exclusion criteria (Table 1). An examination of journal titles and abstracts captured salient studies which were short listed as suitable for inclusion in the literature review. If the prevalence of laxative use was not specifically stated, it was calculated from the published data by dividing the number of participants using laxatives by the total number of participants in the sample and expressing the result as a percentage. This applied to both general community and constipated population samples. Articles reporting laxative use in constipated populations were segmented into chronic constipation and any constipation (self-defined constipation including both chronic and sporadic constipation).

## 3. Results

A total of 31 articles spanning 18 countries met the inclusion criteria. The prevalence of laxative use was reported in 22 studies. In addition, data were provided in 22 studies which enabled the prevalence in either general community or constipation population samples to be calculated (Table 2). Of notable interest, two studies [18,19] reported data for more than one country and one European study reported combined data for 10 countries [14]. In addition, one article [20] described laxative choice and utilisation but did not report any data relating to prevalence of use. The remaining 27 studies reported data for individual countries or regions. Interestingly, only four studies specifically surveyed the usage of laxatives with most studies focusing on constipation rather than laxative use.

### 3.1. Prevalence of Laxative Use

A total of 16 studies surveyed general community populations where the prevalence of laxative use was either reported or calculated. The overall prevalence of laxative use by adults in the community ranged from 1% to 18% in studies conducted in USA [21,22,23,24,25], Europe [5,26,27,28,29,30], Asia [31,32,33] and Brazil [34] (Table 2 and Table 3). However, a definition of laxative was not provided in any of these studies. In addition to these studies, one US study provided a list of laxative products and 75% of participants aged from 40 to 80 years reported that they had used laxatives at some time in their life [35].

The prevalence of laxative use was either reported or calculated in 17 studies of constipated populations. The prevalence of laxative use in samples of constipated adults in the community varied widely from 3% to 87%. An extremely wide range was found for both chronic [9,14,22,23,29,36,38,41,42,45] and any constipation [5,18,19,21,36,37,40,43,46] (Table 2 and Table 3). Only one study clearly defined laxative use and provided a list of products to survey participants [14]; in this European study, the prevalence of laxative use in chronic constipation was 68%. This contrasts with an Australian study where a prevalence of only 3% was reported for undefined laxatives in chronic constipation [45].

In most countries, laxative usage in females was higher than males [5,18,19,39] although male usage was higher than females in the USA, UK and Italy [18]. Laxative use generally increased with age [18,19,39] except in Spain, Korea, China, Indonesia and Brazil [5,19]. Laxative use was associated with lower education and lower income levels in the USA; however, no such associations were found in any other countries [18,19].

### 3.2. Laxative Choice, Utilisation and Satisfaction

The popularity of laxatives chosen by adults to manage chronic or any constipation varied by country and/or region. In North America [9,37,38], fibre/bulk-forming laxatives such as ispaghula were the most popular; stool softeners such as docusate were also popular, and prescription products featured prominently in US studies [9,38]. In Europe and certainly in Italy [14,20], contact laxatives such as senna and bisacodyl, and osmotic laxatives such as lactulose, were more popular than bulk-forming laxatives. Products administered by the rectal route also appeared to be popular in Italy [20].

The involvement of healthcare professionals in laxative product selection appears to be limited. A Spanish study found that only 39.4% of those with self-reported constipation or using laxatives in the last year had sought consultation [29]. An Italian study found that only 58.2% of laxative purchases were made on healthcare professional recommendation [20]; the choice of product was influenced by several sources: doctor (37.7%), pharmacist (20.5%), relatives (14%), acquaintances (12.1%) and advertising (11.7%). A Canadian study reported that 59.5% of adults had attempted self-management of constipation for more than a year before consulting a doctor [37] while in the USA an average of three laxative products are used prior to consulting a healthcare professional [9].

The purpose for using laxatives has not been studied, although it is apparent in some studies that the purpose may not always be for treatment of constipation. For example, in a survey of 1417 adults in Korea [32], the total prevalence of laxative use was 4.7% but only 2.6% of the sample were identified as having chronic constipation according to Rome II criteria. This indicates that 2.1% of adults were using laxatives to either prevent chronic constipation, or treat other forms of constipation, or for some other purpose, such as weight loss [12]. Using laxatives for purposes other than treatment is also evident in UK and Spanish studies where up to 4% of laxative users appear to have no constipation whatsoever [5,39] which suggests that laxatives are either being used successfully for prevention of constipation or being used for some other purpose. Italian researchers were concerned about inappropriate use when they found that that 40% of adults were using laxatives when having three or more bowel motions per week [20]. Usage of laxatives on a daily basis was reported in two studies [5,20].

A high proportion of adults with chronic constipation have reported dissatisfaction with laxatives. A US study reported 47% of 533 adults with chronic constipation were not completely satisfied with laxatives or fibre, 82% of which was related to dissatisfaction with efficacy [38]. Another US study found that, of 1223 adults with chronic constipation, only 40% were satisfied with OTC laxatives [9]. A European study of 793 adults with chronic constipation found that only 28% were satisfied with laxatives used, with 44% being neutral and 28% dissatisfied [14]; satisfaction ratings were similar for all laxative classes.

Adults with any constipation are also dissatisfied with laxatives. In a Canadian study of 200 adults using laxatives for self-reported constipation, 50% were satisfied, 18% were neutral and 32% were dissatisfied [37]. In an Italian study of 7324 laxative purchasers in pharmacies, only 30% purchased the same product that they had purchased in the past, whilst all others chose another product because of “reduced effect” [20]. This indicates that 70% were dissatisfied with the efficacy of laxatives purchased previously.

## 4. Discussion

### 4.1. Prevalence of Laxative Use

There are a number of possible explanations for the wide range of results in studies estimating the prevalence of laxative use. Firstly, any differences in prevalence between countries might be explained by the same factors as differences in prevalence of constipation, i.e., differences in culture, diet, environment and genetics may be partly responsible [8]. For laxatives in particular, socioeconomic differences and differences in healthcare systems may be important considerations as they may impact the availability and affordability of laxative products in different countries. Particular aspects of healthcare systems which may differ between countries include differences in product availability with and without prescription, and differences in product reimbursement.

It is difficult to compare prevalence when different studies have used different study designs. One research group has conducted multinational studies in 11 countries using the same methodology and questionnaire [18,19]. In each country, the sample size was 2000 subjects, aged 15 years or older and representative of the country’s population (except China where the sample size was 2100 and subjects aged over 60 years were excluded). Using the same sample size and data collection method in each country should ensure consistent data and enable comparisons between countries. However, because the term “laxative” was not defined and no list of laxative products was provided, the legitimacy of such comparisons is weakened. Nevertheless, calculation of the prevalence of laxative use in the community shows that prevalence ranged from 16% in Korea to 40% in USA and Indonesia.

Within one country, it might be expected that the prevalence of laxative use would fall within a narrow range, but this has not been the case in the studies reviewed. For example, two Canadian studies have reported different prevalence rates. In a phone survey of 1149 adults with self-reported constipation over three months [36], 34.3% had used laxatives (laxatives were not defined other than the use of prescribed or OTC medication for constipation during the past three months). However, in another Canadian survey 86.5% of 200 participants self-reporting constipation over the last 12 months had used some form of laxative products which included herbal or homeopathic products, fibre and foods [37]. This disparity illustrates that vastly different results may be obtained from the same country when different survey methods, different sample sizes, different constipation definitions, different time periods and no standard laxative definitions are used.

Differences in study design will influence prevalence results. For example, various data collection methods have been used in studies, the most common being face-to face (FTF) interviews. Similar to constipation prevalence studies [8], the research method used may influence survey results. Because of participant embarrassment, FTF interviews may result in lower prevalence rates compared to mail or internet surveys. For example, in North America [18,36,38] and Europe [14,18,29] internet surveys have reported prevalence rates that were up to twice those of surveys conducted by FTF or phone interviews. Another aspect of study design relates to the sample. As with constipation prevalence [8], the sample size may affect the prevalence of laxative use. Study samples have ranged in size from 200 [37] to 72,000 [33] participants. Because sample size calculations have usually not been provided, it is not clear if the chosen sample sizes are appropriate. It is also not clear in most surveys if the sample used was nationally representative; in over half of the studies, regional populations were surveyed. Nationally representative samples are preferred for estimation of prevalence, along with some evidence of representativeness. Similar to constipation prevalence [8], the age range of the sample is another important consideration. In most studies of the general adult population, participants sampled were at least 15 or 18 years old with no upper limit but in some studies [5,21,22,23,31,32,33,39,44], the age of participants was restricted, therefore not all adults in the community were included.

Unfortunately, the majority of studies have not provided definitions of the term “laxative” which means it was self-defined by survey participants. One study of adults with chronic constipation [14] defined the term precisely and included a product list to aid recall; the prevalence was 30% or more higher than most other comparable studies where the meaning was self-defined [22,23,29,36,42,45].

Provision of a product list not only aids definition but also improves recall by providing a useful memory aid [47]. If not defined, it is possible that participants may not regard products such as bulk-forming laxatives and herbal products as laxatives. Also, in some studies where laxatives have not been precisely defined, certain products such as bulk-forming (fibre) products have been either specifically included [22,31,38] or excluded [39]. The ATC laxative definition (A06A: Drugs for constipation) is an international drug classification system, that could be used as a standard definition [11]. The ATC definition includes all OTC laxative agents including bulk-forming laxatives and herbal laxatives, oral and rectal forms, as well as prescription laxatives. In studies reporting the prevalence of laxative use in constipated populations, the definition of constipation is an important consideration as this will also influence the result [8]. Differentiation is usually made between chronic and any constipation. For chronic constipation, most studies used one of the Rome criteria definitions. The majority of studies have reported laxative use with only one definition of constipation.

The recall period used in surveys is an important consideration when estimating prevalence of laxative use [47]. Most studies did not specify any time period. Yet, some studies enquired about current laxative use [9,14,38], and others defined a time period for laxative use such as the past two weeks [27,28], one month [21,26], 3 months [36] or 12 months [18,19,22,23,29,33,37,40]. Clearly the recall period should be defined, and different recall periods will influence the estimated prevalence of laxative usage [47]. Whenever information is elicited from participants, a potential for recall bias exists.

### 4.2. Laxative Choice, Utilisation and Satisfaction

Laxative choice varied by country. In North America, stool softeners such as docusate were popular despite a lack of evidence regarding efficacy [48,49,50], and prescription products feature prominently in US studies [9,38], possibly because more new products have been approved there than elsewhere. An important consideration with laxative choice is the year in which the study was conducted. Many studies were over ten years old and older studies may be less relevant because of changes in product preference and availability. For example, the increasing world-wide use of macrogol as an OTC osmotic laxative and the recent availability of new prescription laxatives in some countries need to be considered [51].

Most adults attempt self-management in the first instance [9,37]. In most cases, healthcare professionals are not consulted [29,36,52] and importantly, healthcare professionals are usually not involved with OTC laxative product selection [20,53]. It has been postulated that this might be the result of advertising and other media as well as the possibility of patient embarrassment in discussing constipation [54]. Consequently, OTC laxative choice and use may not always be appropriate [20]. Without advice from healthcare professionals, appropriate product selection and directions for use are challenging for the consumer [53] who may be influenced by other less reliable sources of information [20] such as advertising, acquaintances, or relatives.

High levels of dissatisfaction with laxatives have been reported mainly because of poor efficacy with no differences noted in laxative classes. This may be related to how laxatives are being used. Daily use of laxatives indicates use for prevention rather than treatment. Another indication of preventive use is that some adults report laxative use but not constipation. It seems clear that there is a dual purpose for laxative use—prevention and treatment of constipation, apart from any use not related to constipation. However, no studies have investigated this aspect. In particular, no studies have assessed the perceived effectiveness of laxative agents used for treatment compared to those used for prevention of constipation. Appropriate OTC laxative choice for the intended purpose should be based on the onset of action [55]. The high levels of dissatisfied laxative users in several studies suggest that laxatives are not being used appropriately [9,14,37,38]. Knowledge of the effectiveness of laxatives in practice is essential for improving the management of constipation in the community.

### 4.3. Limitations

A limitation of the literature review is the risk of bias, whereby the studies included were conducted in an English-speaking context and written in English and were further refined according to the inclusion/exclusion criteria. The risk of bias is acknowledged since some relevant studies may have been excluded from the literature reviewed. The authors also acknowledge potential recall bias because survey results were based on recall of participants, the period of which varied in different studies. Furthermore, differences in healthcare systems in different countries will also influence the results obtained in different studies.

## 5. Conclusions

It is difficult to determine the true prevalence of laxative use in the community. Estimates of laxative prevalence in community-dwelling adults vary greatly, whether in general community populations or in constipated populations. Apart from any country differences, a number of other factors may explain the wide variation. One important factor is the lack of a precise laxative definition in most studies which makes it difficult to determine what agents have been included or excluded. Other factors to consider are different recall periods, study designs and sampling differences. For studies in constipated populations, different constipation definitions also affect laxative prevalence estimates.

To estimate the prevalence of laxative use more accurately, an internationally accepted laxative definition such as the ATC definition, a specified recall time period, a nationally representative sample of appropriate size and a questionnaire which includes a list of laxative product names to facilitate recall are recommended. In constipated populations, it is recommended that universally accepted constipation definitions are used such as the Rome criteria for chronic constipation, or self-reported constipation in a specified time period for any constipation.

Few published studies have investigated the choice and utilisation of laxatives, and whether users are satisfied with their use. It appears that laxatives are not always being used for treatment of constipation and that they are also used for prevention of constipation. This distinction in the purpose of laxative use requires investigation along with the sources of influence for choice of laxative. It seems that healthcare professionals are not always involved in laxative choice, but this also needs to be further researched particularly with regard to the dual purpose of laxative use. Studies regarding laxative choice are now outdated and new studies investigating currently available laxatives are required, particularly to assess their effectiveness in preventing and treating constipation in the community.

To improve constipation management in community and primary healthcare settings, knowledge of the true prevalence and utilisation of laxative use is required, and this review indicates the need for further research in these areas.

## Figures and Tables

**Table 1:** Literature inclusion and exclusion criteria.

Category	Inclusion	Exclusion
Population Sample	Community-dwelling adults (aged 15 and above)	Not residing in the community
	General population and/or constipated population	Sample size fewer than 100 participants
	Sample size greater than 100 participants	Samples of subpopulations e.g., only one gender or samples of older populations
Variables	Prevalence of laxative use	No data regarding laxative use
	Laxative utilisation	
	Self-defined constipation	
	Chronic constipation	
Period	1989–2019	Prior to 1989
Linguistic Range	English language	Non-English language
Type of Study	Population-based surveys	Qualitative studies
	Cross-sectional surveys	

**Table 2:** Range of prevalence of laxative use in community-dwelling adult populations.

Community-Dwelling Adults (General Population)			Community-Dwelling Adults Reporting Chronic Constipation		Community-Dwelling Adults Reporting Any Constipation			
No. of Studies Reporting Prevalence	No. of Studies Where Prevalence Calculated from Data	Prevalence Range	No. of Studies Reporting Prevalence	No. of Studies where Prevalence Calculated from Data	Prevalence Range	No. of Studies Reporting Prevalence	No. of Studies where Prevalence Calculated from Data	Prevalence Range
7	9	1 to 18%	9	2	3 to 72%	6	11	8 to 87%

**Table 3:** Prevalence of laxative use in studies of community-dwelling adult populations.

Author (Reference)	Location	Sample Size	Age Range (Years)	Method	Laxative Definition	Time Period (months)	% Prevalence of Use—General	% Prevalence of Use—Constipated	Constipation Definition
Everhart 1989 [21]	USA (NHANES I)	5951	35–84	FTF interview	ND but included stool softener	Monthly or more	17.9 *	58.8 *	Self-report (NTP)
Talley 1991 [22]	USA (Olmsted)	835	30–64	Mail survey	ND but included bran & bulking agents	12	15.6 *	42.9	CC (BDQ)
Talley 1993 [23]	USA (Olmsted)	690	30–64	Mail survey	ND but included enemas	12	6.8 *	20	CC (Rome I)
Harari 1996 [24]	USA (NHIS)	42,375	18+	FTF interview	ND but included stool softener	Monthly or more	11.5	NR	N/A
Wald 2008 [18]	USA	2000	15+	FTF interview	ND	12	NR	40.2 *	Self-report (12 months)
Pare 2001 [36]	Canada	1149	18+	Phone survey	ND	3	NR	34.3	Self-report (3 months)
								26.3	CC (Rome II)
Ferrazzi 2002 [37]	Canada	200	18+	Phone survey	ND but included herbal, homeopathic, fibre & foods	12	NR	86.5	Self-report (12 months)
Choung 2012 [25]	USA (Olmsted)	2853	20+	Mail survey	Laxative (ND) or fibre	12	13.7 *	52	Persistent CC
								28	Non-persistent CC
Johanson 2007 [38]	USA	553	18+	Internet survey	Laxative (ND) or fibre	Current	NR	72	CC (Rome II mod)
Roberts 2003 [35]	USA (North Carolina)	1651	40–80	FTF interview	All laxatives including fibre	Lifetime	70.9 (cancer)	NR	N/A
							74.6 (controls)		
Harris 2017 [9]	USA	1223	18+	Internet survey	All laxatives including fibre	Current	NR	40 (OTC)	CC (Rome IV or doctor diagnosed)
								16 (Rx)	
Heaton 1993 [39]	UK (East Bristol)	1892	25–69 (females) 40–69 (males)	FTF interview	ND excluding bulking agents and bran	NTP	NR	46.2	Self-report (Often/always)
Siproudhis 2006 [40]	France	7196	15+	Mail survey	ND	12	NR	35.3	FDD
								43.4	OC
Enck 2016 [30]	Germany	15,002	18+	Phone interview	ND	NTP	4.4	NR	N/A
Wald 2008 [18]	UK	2000	15+	FTF interview	ND	12	NR	35.8 *	Self-report (12 months)
Wald 2008 [18]	France	2000	15+	FTF interview	ND	12	NR	32.9 *	Self-report (12 months)
Wald 2008 [18]	Germany	2000	15+	FTF interview	ND	12	NR	30.8 *	Self-report (12 months)
Wald 2008 [18]	Italy	2000	15+	FTF interview	ND	12	NR	34.6 *	Self-report (12 months)
Bassotti 2004 [26]	Italy	298	Adults (ages not reported)	Written questionnaire	ND	1	5	NR	N/A
Galvez 2006 [5]	Spain	349	18–65	Mail survey	ND (but including suppositories & enemas)	NTP	14	40	Self-report (12 months)
Rey 2014 [29]	Spain	1500	18+	Phone interview	ND	12	11.3	38.9 *	CC (Rome III)
Carrasco-Garrido 2008 [27]	Spain	19,514	16+	FTF interview	ND	2 weeks	1.16 *	NR	N/A
Carrasco-Garrido 2010 [28]	Spain	30,428	16+	FTF interview	ND	2 weeks	3.0 *	NR	N/A
Muller-Lissner 2013 [14]	Europe (10 countries)	1255	18+	Internet survey	All classes including fibre and rectal products	Current	NR	68	CC (Self-report)
Jeong 2008 [32]	Korea	1417	18–69	FTF interview	ND	Current	4.7 *	NR	N/A
Wald 2008 [18]	Korea	2000	15+	Phone survey	ND	12	NR	16 *	Self-report (12 months)
Wald 2010 [19]	China	2000	15–60	Phone survey	ND	12	NR	39 *	Self-report (12 months)
Wald 2010 [19]	Indonesia	2000	15+	FTF interview	ND	12	NR	40 *	Self-report (12 months)
Adibi 2007 [31]	Iran	995	14–41	Written questionnaire	ND including bulking agents	NTP	7.7 *	NR	N/A
Herz 1996 [41]	Israel	531	21+	Written questionnaire	ND including suppositories	NTP	NR	41	Self-report (NTP)
Rooprai 2017 [42]	India	925	18+	FTF interview	ND	NTP	NR	40.1 *	CC (Rome lll)
Kubota 2016 [33]	Japan	72,014	40–79	Written questionnaire	ND	12	10.5 *	NR	N/A
Tamura 2016 [43]	Japan	5155	20–79	Internet survey	ND (OTC)	NTP	NR	7.9	Self-report (NTP)
Song 2019 [41]	China	6318	18+	Internet survey	ND	NTP	NR	25.2	CC (Self-report)
Wald 2008 [18]	Brazil	2000	15+	FTF interview	ND	12	NR	21.9 *	Self-report (12 months)
Wald 2010 [19]	Argentina	2000	15+	FTF interview	ND	12	NR	31.9 *	Self-report (12 months)
Wald 2010 [19]	Colombia	2000	15+	FTF interview	ND	12	NR	28.4 *	Self-report (12 months)
Chinzon 2015 [34]	Brazil (Sao Paulo)	3028	18+	Phone interview	ND	NTP	13.4	NR	N/A
Lynch 2001 [44]	New Zealand (Canterbury)	717	18–70	Mail survey	ND	NTP	4.7	NR	N/A
Ng 2014 [45]	Australia (Western Sydney)	396	18+	Written questionnaire	ND	NTP (regular use)	NR	3.1	CC (Rome III modified)

Notes: * Calculated from data published; NR = not reported; ND = not defined; CC = chronic constipation; FTF = face to face; BDQ = bowel disease questionnaire; OTC = over the counter; Rx = prescription; NTP = no time period reported; N/A = not applicable; + = greater than or equal to.
